# Anesthetic Management of a Patient With Pierre Robin Sequence: A Case Report

**DOI:** 10.7759/cureus.83687

**Published:** 2025-05-07

**Authors:** Joshua Singavarapu, Sunny Yoo, Vitaliy Borodulin, Galina Borodulina

**Affiliations:** 1 Anesthesiology, SUNY Downstate Medical Center, Brooklyn, USA; 2 Anesthesiology, Private Practice, New York, USA; 3 Anesthesiology, Northwell Health Staten Island University Hospital, Staten Island, USA

**Keywords:** general anesthesia, patient safety, pediatric anesthesiology, pediatric surgery, pierre-robin sequence

## Abstract

Pierre Robin Sequence (PRS) is a rare congenital disorder characterized by a triad of micrognathia, glossoptosis, and airway obstruction. This case report presents a rare instance of PRS marked by congenital airway obstruction, which significantly complicated endotracheal intubation with vocal cord motion and a smaller airway to navigate. It will describe what can be expected from such airways and the approaches taken to manage them. The patient was a five-month-old female with PRS, undergoing removal of bilateral mandibular distractors for treatment of micrognathia. Preoperative evaluation revealed a well-nourished, full-term, American Society of Anesthesiologists (ASA) Class II infant in no distress, breathing spontaneously on room air with previously placed bilateral mandibular distractors. Anesthesia induction utilized 8% sevoflurane in 100% oxygen, maintaining spontaneous ventilation. Video laryngoscopy showed a large U-shaped cleft palate and a Cormack-Lehane class II view. The uneventful procedure included dexamethasone, acetaminophen, ketorolac, propofol, and local anesthesia (lidocaine with epinephrine and marcaine). Total IV fluids were 350 ml, blood loss was minimal, and extubation was successful with stable recovery. Video documentation of the intubation sequence is included within this report. This report demonstrates effective airway management strategies tailored to PRS-related anatomical challenges and highlights the vital role of anesthesiologists in clinical decision-making teams for the management of complex airway scenarios.

## Introduction

Pierre Robin Sequence (PRS) is a rare congenital disorder, with an incidence ranging from 1 in 8000 to 1 in 14,000 newborns [1]. It presents with a classic triad of micrognathia (small mandible), glossoptosis (posterior-inferior retraction of the tongue in the pharynx), and airway obstruction [2]. Ninety percent of patients with PRS have a cleft palate, as was the case in this patient, though it is not part of the diagnostic criteria [3]. Neonates with PRS typically present with signs of respiratory distress, feeding difficulties, aspiration, and failure to thrive [4]. The pathologic events leading to PRS are not fully understood, but extensive involvement with over 40 clinical syndromes has been documented. The high likelihood of concomitant congenital diseases results in clinical variation in the presentation of PRS.

From an anesthesiologist's perspective, PRS presents with a difficult airway. Though there are many airway management protocols that have been evaluated, the gold standard for intubation of PRS patients is fiberoptic-assisted intubation [5]. Additionally, a flexible nasolaryngoscopy can be used to evaluate the degree of airway obstruction pre-operatively. In order to relieve this airway obstruction, the patient can be placed in the prone position to allow the mandible and tongue to fall forward and reduce airway obstruction [3]. Additionally, a tongue suture can be placed to pull the tongue forward in the supine position. Maintenance of spontaneous ventilation is desirable until the airway is secured. This case report presents a rare instance of PRS marked by congenital airway obstruction, which significantly complicated endotracheal intubation. It describes what can be expected from such airways and the approaches taken to manage them.

## Case presentation

The patient was a five-month-old full-term female. She was diagnosed with PRS and a large U-cleft palate at birth. After delivery, the patient was in the neonatal ICU (NICU) for two weeks in the lateral position to relieve airway obstruction. At age six weeks, the patient had bilateral mandibular distraction osteogenesis for the treatment of micrognathia. At the age of 5 months, she presented for the removal of bilateral distractors. Preoperative evaluation revealed a well-nourished child, breathing spontaneously on room air, with a large cleft palate and mandibular hardware and an unremarkable cardiopulmonary exam. She weighed 7.2 kg and had stable vital signs. The patient was classified as American Society of Anesthesiologists (ASA) Class II. Although the patient was presenting for removal of the mandibular distractors, suggesting prior airway optimization, the anesthetic plan still accounted for potential airway challenges due to underlying craniofacial abnormalities. General anesthesia with inhalation induction and maintenance of spontaneous ventilation was chosen to preserve spontaneous breathing in case of difficult intubation. Tracheal intubation was performed under controlled conditions, and a pediatric ENT surgeon remained on standby for emergency surgical airway access.

Anesthesia was induced with 8% sevoflurane in 100% oxygen with maintenance of spontaneous ventilation. Eyes were taped. A 22-gauge IV was placed in the right saphenous vein. Glycopyrrolate 0.1 mg and fentanyl 5 mcg IVP (IV push) were given. Video laryngoscopy with Miller 1 blade revealed a large U-cleft palate and a class II Cormack-Lehane view of the vocal cords. Spontaneous ventilation with vocal cord motion was observed (Video 1). Two attempts at laryngoscopy were performed (one resident and one attending) with successful placement of a 3.5 cuffed endotracheal tube (ETT). The attending's successful placement was recorded in Video 1, with the difference being that the attending was able to maneuver the ETT past the vocal cord motion into the trachea. The ETT cuff was inflated with 0.5 ml of air and secured at 12 cm at the lips, with equal bilateral breath sounds confirming placement. Vital signs were stable throughout intubation.

**Video 1:** Successful Intubation of Pierre Robin Sequence Patient

The procedure ensued uneventfully, with the patient receiving dexamethasone 3 mg, acetaminophen 110 mg, ketorolac 3.6 mg, and propofol 5 mg throughout the case. Local anesthesia was administered by the surgeon, lidocaine 0.5% with epinephrine and bupivacaine 0.25%. Total IV fluid was 350 ml. The patient was extubated successfully and taken to the post-anesthesia care unit (PACU) in stable condition. Vitals in the PACU showed an axillary temperature of 97.5 F, heart rate (HR) of 146 bpm, BP of 80/54, respiratory rate (RR) of 20 breaths per minute, and O2 saturation of 100% on room air.

## Discussion

This case report highlights the anesthetic management for PRS patients, emphasizing through the supplemental video how airway intervention may be difficult because of their congenital defects. Additionally, due to the young age of many PRS patients who present to the OR, it is important to consider anatomic and physiologic differences in this age group that may affect the choice of anesthetic technique. Sevoflurane was used for the induction of anesthesia to maintain spontaneous ventilation as a precautionary measure, given the patient’s history of a difficult airway. Although the mandibular distractors had been placed, residual airway compromise can persist due to underlying craniofacial abnormalities. Preserving spontaneous ventilation during induction allowed for greater safety in case of unexpected difficulty with mask ventilation or intubation, in accordance with pediatric difficult airway management principles. Opioids were used sparingly as studies demonstrate that patients with PRS may have opioid sensitivity secondary to chronic airway obstruction and hypoxia [6]. This patient had mandibular distractors in place to partially correct micrognathia, improving airway patency [7] while not obstructing her airway on induction of anesthesia. Her anesthetic was expertly calibrated with safeguards in place, such as spontaneous ventilation on induction, ENT surgeon availability, and video laryngoscopy for intubation [8]. These safety measures ensured a smooth anesthetic course and successful extubation inside the OR. Understanding the variety of PRS presentations across age groups will help to individually tailor anesthetic techniques for each patient in this population.

Intubating infants in a similar patient category of younger than one year old also presents considerable anatomical and physiological complexities, necessitating a methodical and individualized approach. Recognizing distinct features such as an enlarged occiput, an anterior larynx, a larger tongue, and a floppy epiglottis guides careful selection of airway management strategies [9,10]. Rapid desaturation further underscores the importance of meticulous preparation, pre-oxygenation, and swift yet precise intervention. Reviewing historical intubation records - including hospital information systems dataon prior airway difficulties and sedation responses - is essential to inform the selection of appropriate techniques. Adherence to the pediatric difficult airway algorithm [11] ensures structured, progressive escalation, optimizing patient safety by systematically preparing backup techniques and resources, starting from patient airway assessment to the use of airway devices. It speaks on the stepwise manner in which cases should be approached and is an appropriate standard of care that was followed for this patient.

## Conclusions

This case report demonstrates how the systematic perioperative evaluation of a patient with PRS remains critical, even after initial airway correction with mandibular distraction. Although the primary anatomical cause of airway obstruction had been addressed, patients with PRS often retain residual craniofacial abnormalities that can continue to pose airway management challenges. In this case, tailored anesthetic strategies, including maintenance of spontaneous ventilation and readiness for alternative airway approaches, contributed to a smooth intubation and perioperative stability. Highlighting such evaluations helps emphasize that prior surgical intervention does not eliminate the need for careful airway planning. Given the rarity and variability of PRS presentations, sharing successful anesthetic management approaches contributes to the broader understanding and preparedness needed for safe and effective care in future cases.
